# Anticorrosion Behavior of Zeolite Coatings Obtained by In Situ Crystallization: A Critical Review

**DOI:** 10.3390/ma12010059

**Published:** 2018-12-24

**Authors:** Luigi Calabrese

**Affiliations:** Department of Engineering, University of Messina, Contrada di Dio, 98166 Messina, Italy; lcalabrese@unime.it

**Keywords:** corrosion resistant coatings, zeolite, functional coatings, in situ crystallization, hydrothermal

## Abstract

Zeolites are crystalline nanoporous aluminosilicates. Thanks to their intrinsically nanoporous structure they are widely used as molecular sieves, for exchanging ions, or, also thanks to the high surface area of these structures, for catalytic applications. Furthermore, thanks to their thermal and chemical stability, in recent years zeolite coatings have been evaluated for application as anti-corrosion coatings. The non-toxicity of this class of coatings makes it possible that they will be an environmentally friendly alternative to conventional chromate-based coatings. This article provides a brief review of the anti-corrosion performance of zeolite coatings, applied by direct synthesis technique to several metals and alloys, as discussed in the literature. After a short description of the microstructure and properties of zeolites, the discussion addresses the research activities related to this topic, as reported in the literature. Comparative analysis of literature results supported the dry-gel conversion method as a promising approach that combines a simplified synthesis procedure with anti-corrosion coating performance. Based on these considerations, an evaluation of future trends is discussed along with the final remarks.

## 1. Introduction

The development of coatings with effective active or functional properties for metallic substrates is a stimulating research topic in the field of zeolites, which could lead to new and innovative prospective applications. In fact, zeolite based coatings have been, in recent years, proposed for applications in various fields including but not limited to catalytic reactors [1], molecular decontamination [2], water desalination [3], for improving the heat exchange in adsorbers for adsorption heat pumps [4], for added antimicrobial actions in biomedical applications [5,6], or to be used as anti-corrosive coatings for uses in severe environmental conditions [7,8]. The very good thermo-chemical stability of zeolites allows their application in critical and aggressive environments. Furthermore, their intrinsic non-toxicity also represents a valid possibility that zeolite films may be used as an interesting, environmentally friendly alternative to surface passivation treatment based on chromates, allowing potential expansion of this new green class of coatings into particularly restrictive industrial sectors such as the food or pharmaceutical industries [9,10]. However, the feature that adds most value to the potential use of zeolite coatings is its capacity to host extra ions and molecules in its regular porous structure. Such molecules can be exchanged with the environment or can act directly incorporated in the coating, for example as corrosion inhibitors. In such a context, zeolite coatings could be classified as ‘smart coatings’ as they are able to react specifically to external stimuli from the surrounding environment in which the coated components work during their service life.

A large number of types of zeolites, both natural and synthetic (X, Y, ZSM-5, …), have been deposited on several substrates in order to give added function to the component on which it was deposited. Several industrial purposes can be identified, ranging from separation and catalysis processes [11,12], to adsorption [13,14] or sensing [15,16] technology.

**Figure 1:**
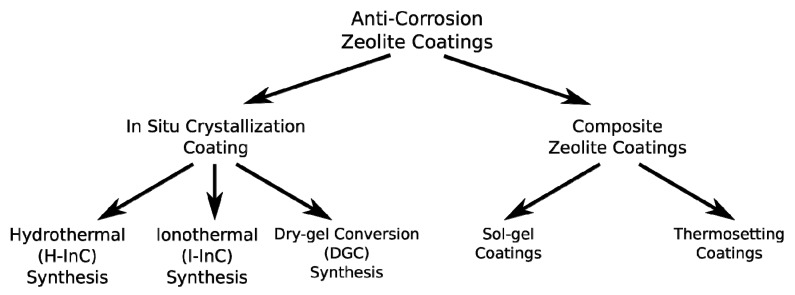
Synthetic scheme of the anti-corrosion zeolite coating techniques.

Depending on the support and purpose of use, different deposition techniques can be identified as suitable for anti-corrosion zeolite metal coating [17,18]. Figure 1 summarizes the synthesis techniques, grouped in two main families: in situ crystallization coatings and composite zeolite coatings. In the former, a direct synthesis of zeolite in the metal substrate is obtained. In the latter, the zeolite is used as a filler of a matrix with layer capabilities.

Among these, in situ crystallization and sol-gel coating are the most widespread. Concerning the sol-gel coating technique, the matrix acts as binder of previously synthesized zeolite crystals, obtaining a composite coating with effective adhesive and cohesive performances. This technology has the advantage of a relatively simple and versatile production process, allowing easy thickness control and scale-up (depending on the component size to be coated).

On the other hand, in situ crystallization involves the direct synthesis of zeolite crystals on the metal support, allowing optimal zeolite/metal adhesion. This allows coatings to be obtained with very high adhesion and cohesion. A potential limitation of this technique is difficulty in scaling up, although some authors have recently realized and characterized an entirely coated exchanger [19,20]. Nevertheless, the potential of this deposition technique for obtaining anti-corrosion zeolite coatings is high, and the experimental results found in the literature are encouraging on this regard.

Consequently, the present review focuses on the zeolite coatings obtained by in situ crystallization. According to the scheme in Figure 1, the different synthesis techniques proposed in the literature are evaluated and critically compared.

This article is structured to provide a brief review on anti-corrosion zeolite coatings. After a short description of the microstructure and properties of zeolites, the discussion is addressed to the research activities, reported in the literature, related to this topic. The Section 1 is focused on zeolite coatings obtained by in situ crystallization synthesis, applying hydrothermal, ionothermal, and dry-gel conversion techniques. In the second part, anti-corrosive aspects of zeolites coatings are discussed, followed by a comparison of the anti-corrosion efficiency of in situ crystallization techniques. Finally, Section 3 assesses the use of zeolite as nanocontainer filler for the development of active smart coatings.

The outcome is a rational, structured research statement based on appraisal of literature results, with emphasis on the limitations and implications of key findings on anti-corrosion performances among different in situ crystallization synthesis methods.

## 2. Microstructure of Zeolites

Zeolites are hydrated silicate-aluminates of alkaline and/or alkaline-earth metals which, structurally, belong to the class of tectosilicates (three-dimensional tetrahedral structured silicates). The zeolitic structure can be represented by the following minimum formula:

Me_x/z_ [Al_x_Si_(1−x)_O_2_]∙wH_2_O (1)

where Me represents one or more cations of valence z; and w is a variable according to several parameters (type of zeolite, the Si/Al ratio, the cationic composition, the temperature, and the partial pressure of water vapor in the environment).

The value of Si/Al is in the range [1;+∞] since, as foreseen by the Loewenstein rule, two contiguous tetrahedra cannot both be centered on an aluminum atom [21].

The structure of the zeolites can be described as a three-dimensional lattice of tetrahedra, the centers of which are occupied by silicon or aluminum atoms and the vertex by oxygen atoms, which act as a bridge between adjacent tetrahedra.

The three-dimensional structure is characterized by cavities and channels, usually occupied by water molecules and extra-reticular cations, which are normally exchangeable. The canals are sufficiently wide to allow the passage of guest species.

**Figure 2:**
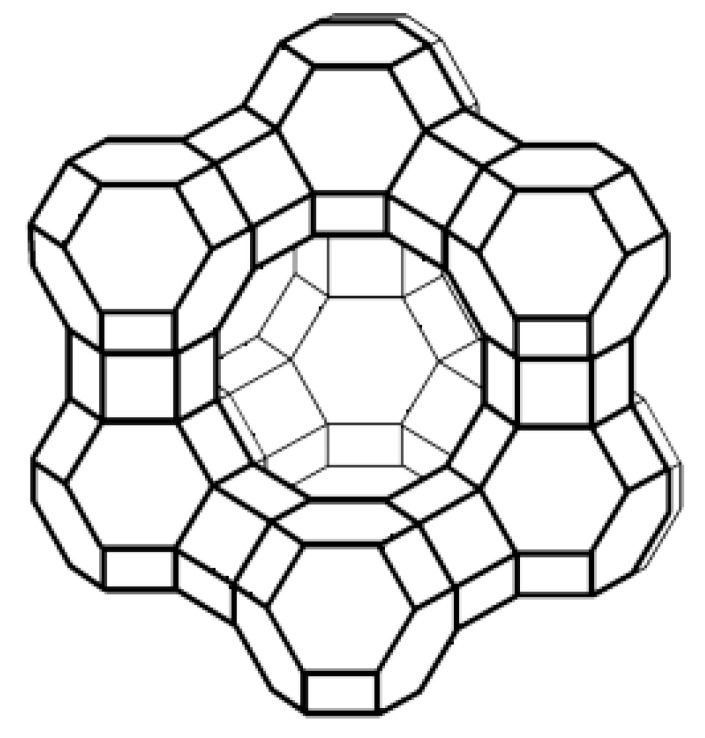
Scheme of the structure of zeolite Y.

In Figure 2, as reference, a scheme of the tridimensional structure of a zeolite Y is reported. The zeolitic structure consists of uniform, intercommunicating cavities and channels (with dimensions between 3 and 10 Å), resulting in a high surface area and large internal volume. These pores, called micropores for their size (<20 Å), are occupied under normal conditions, as seen, by water molecules and metal cations, which are not structural components of the rigid alumino-silicate structure and can be removed or exchanged without altering the stability of the structure itself. This justifies the extraordinary properties that make zeolites widely used as adsorbents, catalysts, and ion-exchange agents. A further, widely applied application is their use as separation membranes by exploiting their molecular sieve effect. At the same time, other interesting applications have been evaluated in the literature for their further use in new industrial fields e.g., dielectric films [22,23], antimicrobial coatings [24,25,26], and heat pumps [27,28].

During the last fifteen years, studies of the anti-corrosive properties of zeolite based coatings have been conducted. The idea of corrosion resistant zeolite coatings arises from possible application advantages such as non-toxicity (useful for very restrictive applications such as food and drugs) and thermal and chemical stability (for severe environmental applications). This makes zeolite a potentially valid structural material for developing high efficiency protective coatings.

## 3. Anti-Corrosion Zeolite Coatings

The first research on the use of zeolite for the production of anti-corrosive protective coatings may be associated with the activity of Yan group [29,30]. In these early works, the zeolite coating was obtained by direct synthesis on an aluminum alloy support. Electrochemical impedance and polarization tests showed, respectively, a high impedance magnitude at low frequency (10^9^ Ohm cm^2^) and a reduction of the corrosion current by 4 orders of magnitude compared to the uncoated alloy. These promising results, supported by good adhesion and stability of the coating, have allowed researchers to hypothesize that such coatings could be developed as environmentally friendly, alternative corrosion-resistant coatings for aluminum alloys. About ten years later, the use of zeolites as barrier or functional coatings became a relevant topic for new research groups which, on the basis of the results achieved by Yan, identified their work as functional [31] or barrier [32,33] coatings for specific applications. The research developments in these years were focused on assessing the anticorrosion capabilities of zeolite based coatings on different metal substrates, such as stainless steel, aluminum, titanium, or magnesium alloys, and evidenced effective protective action of this class of coating. The possibility of obtaining zeolite coatings using the sol-gel technique, instead of direct hydrothermal synthesis, was proposed. This made it possible to diversify and make flexible the surface engineering design for this class of coating. This activity was finalized with the creation of functional coatings for which zeolite has been used as a nano-container to enhance both the inhibiting as well as the barrier properties of the coating [34]. More recently, a dry gel conversion synthesis technique was proposed as a potentially effective approach for obtaining suitable anti-corrosion coatings [35,36]. The idea was to maintain the advantages of coating deposition obtained by sol-gel technique, combined with stability and durability observed for hydrothermal zeolite coatings. These last results make in situ crystallization a flexible and functional industrial solution, and support future functional developments of the coating itself. In particular, in recent years, research and development activities on anti-corrosion zeolite-based coatings have been very intense. Many works have been carried out in order to evaluate and validate the performance of pure and composite zeolite coatings in order to optimize the product, involving different synthesis techniques and matrix types [8,37,38,39,40], indicating that the times are mature for its use in the industrial field.

### 3.1. In Situ Crystallization of Zeolite Coatings

As previously discussed, the direct synthesis and the anti-corrosion performance of high silica zeolite (HSZ) MFI on aluminum substrate was first evaluated by Yan and coworkers [29,30,41].

During in situ crystallization (InC), the zeolite coating crystallizes directly at the metal-liquid interface. In situ crystallization is able to coat complex geometries and narrow and confined cavities, making it a desirable coating approach for irregularly shaped components. Different in situ crystallization approaches have been proposed: hydrothermal (H-InC) [29,30,41], ionothermal (I-InC) [42,43], and dry-gel conversion (DGC) [44,45] crystallization methods.

In Table 1, a brief summary of some scientific articles focused on the anti-corrosion performances of zeolite-based coatings obtained by hydrothermal and ionothermal in situ crystallization techniques is reported. In particular, the main information related to coating typology, synthesized in order to evaluate their effectiveness as a protective coating against corrosion, is indicated. The table shows that most of the supports on which zeolite coatings have been made were an aluminum alloy. The performance of the coating has been investigated in multiple environmental conditions, ranging from chloride environments up to acidic [46] or strongly alkaline (pH 12.5) [47] electrolytes. Coatings based on zeolite have been found to have an effective anticorrosive behavior, with a reduction of the corrosion current by up to 5 orders of magnitude compared to the uncoated support.

### 3.2. Hydrothermal In Situ Crystallization (H-InC) Method

**Figure 3:**
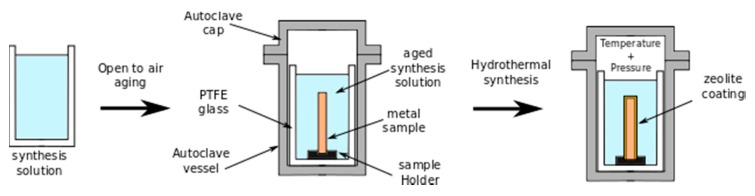
Scheme of hydrothermal synthesis process.

The principle of hydrothermal synthesis is based on the tendency of amorphous silico-aluminate systems to evolve, in an alkaline environment and in hydrothermal conditions, towards tectosilicatic phases, in particular a zeolite type structure. Hydrothermal synthesis is carried out by reacting, at a set temperature and under constant agitation, the weighed quantities of the various reagent compounds in a perfectly sealed autoclave, thus achieving conditions of Autogenic pressure. Alkaline silicate and aluminate solutions or suspensions of colloidal silica with amorphous aluminum hydroxide in alkaline solutions are commonly used as reagents. After mixing, the solution constituents start to gelate. The factors that influence the chemical reaction are generally the temperature and the pressure. Based on this consideration, the hydrothermal synthesis process is usually completed in a sealed autoclave. Figure 3 shows a scheme of the process, consisting of two stages: a first stage, where aging of the solution occurs with the formation of a gel starting from the synthesis solution. Afterwards, the aged solution is placed inside a Polytetrafluoroethylene (PTFE) sealed autoclave in order to activate the in situ crystallization of the zeolite. The sample is located inside the autoclave in order to have its surface in direct contact with the solutions. The germination of zeolite crystals will start at the metal surface, favoring the formation of thick and well adherent zeolite coatings with increasing time.

Cheng et al. [29] and Beving et al. [51] evaluated the H-InC coating process on several aluminum alloys (AA2024-T3, AA5052-H32, AA6061-T4 and AA7075-T6), evidencing the formation of a uniform and compact high silica zeolite (HSZ) layer with promising anti-corrosion performances in a wide pH range electrolyte solutions, from acidic to alkaline ones. Similar results were observed by Mitra et al. [41], where zeolite coatings were synthesized by hydrothermal synthesis method on aluminum (Al-2024-T3 and Al-6061-T4) and stainless steel (SS-304) alloys, evidencing good adhesion and electrochemical stability in acidic and alkaline media.

Banerjee et al. [49] investigated the anti-corrosion performances of a ZSM-5 zeolite coating deposited on AZ91D magnesium alloy using the in situ crystallization approach. The results evidenced that, although the magnesium alloy was a metal substrate with relevant electrochemical reactivity in severe environmental conditions, as in hydrothermal synthesis, a zeolite coating with suitable adhesion, homogeneity, and barrier action could be obtained. In particular, they observed that the corrosion resistance of the zeolite coated sample in 0.1 M NaCl solution was about one order of magnitude higher than the uncoated one. At longer immersion times (after 168 h of immersion) the difference increased up to at least five order of magnitude. In sodium chloride solutions, the high efficiency of zeolite layers can be justified, considering that zeolite can improve the resistance of chloride ingress [52,53]; the chloride ions have greater difficulty interacting with the zeolite framework, making it difficult to reach the metal substrate. Eventually, the depassivation phenomenon is delayed or inhibited.

However, low silica zeolite (LSZ) coatings also represent a relevant class of materials, effective for application in industrial fields. Thanks to their combination of hydrophilicity, adsorption performances, and heat transfer properties [54], the LSZ coatings offer reliable performance in air conditioning systems for residential or industrial applications [13,55].

However, the strongly aggressive synthesis conditions have severely limited its applicability on corrosion-sensitive substrates such as aluminum alloys. In this context, several research approaches have been proposed to deposit LSZ coatings on aluminum substrates by direct synthesis, highlighting process difficulties. The synthesis process of low silica zeolites (such as LTA, X, or Y) is characterized by a very high pH of the solution (often higher than 14). It cannot be performed directly on aluminum, as it results in a rapid dissolution of aluminum alloys during the initial synthesis phases [56], while it can be applied in order to obtain LSZ coatings on stainless steel [57].

To overcome such a limitation on aluminum substrate, a possible approach is to pre-treat the surface in order to grow a stable zeolite layer in the specific synthesis conditions (e.g., MFI synthesis can be performed at a pH not aggressive for aluminum). Munoz et al. [56] proposed a three step method on an Al 2024-T3 substrate. In the first step, a protective high silica zeolite (HSZ) ZSM-5 layer was applied on the aluminum substrate. In the second step, a bridging layer constituting a combined ZSM-5 and zeolite Y film was applied. Finally, the low silica zeolite (LSZ) Y film was synthesized on the top. Analogously, in Reference [54] a two layer deposition was proposed. The bottom layer was a high silica zeolite (HSZ) MFI coating, formed directly on the aluminum alloy by in situ crystallization. Finally, the top layer was a LSZ (zeolite A) coating. Furthermore, Calabrese et al. [32] proposed a hydrothermal single step synthesis procedure for zeolite Y directly on aluminum substrate by using an amine template (triethanolamine—TEA). The proposed approach retained both the relevant advantages from a technological point of view, without losing mechanical stability, and corrosion durability of the zeolite coating in severe environmental conditions. The optimization of the complex in situ crystallization process characterized by several synthesis parameters is a key point to in creating a coating with high mechanical properties as well as good barrier capabilities and durability for applicability in industrial applications (e.g., chemical industry, biomedical applications, or air conditioning systems) [46,58,59].

Lauridant et al. [60] evidenced the influence of several synthesis parameters (i.e., substrate cleaning, crystallization time, aluminum content in solution, and aluminum substrate nature) on ZSM-5 coating properties, such as Si/Al molar ratio, thickness, and homogeneity. Homogeneity and a lack of cracks or pinholes are relevant aspects that need to be guaranteed for the optimal performance of corrosion resistance zeolite based systems.

Analogously, Wang et al. [61], assessing MFI zeolite coatings growth on AISI 304 stainless steel by hydrothermal synthesis, showed that crystal morphology and orientation can be controlled by a correct optimization of the synthesis composition. In particular, well oriented MFI zeolite layers were obtained in medium OH^−^/Si (e.g., OH^−^/Si = 0.32) and low Na^+^/TPA^+^ ratios (e.g., Na^+^/TPA^+^ < 0.31).

Furthermore, Calabrese et al. [47] evidenced that the synthesis time and drying temperature parameters significantly affected the coating performances. In particular, the protective action suffered by the zeolite layer was enhanced at low drying temperatures, indicating that low temperature favors a more effective protective action of zeolite coating. This behavior was ascribed to the TEA compound entrapped in the zeolite structure. At the same time, synthesis time influenced the coating morphology, homogeneity, and stability. Long synthesis times favored the formation of thick, cracked zeolite layers, characterized further by low adhesion with the metal substrate.

This behavior in hydrothermal synthesis can be justified, considering that the zeolite channels in the ordered structural porosity are partially obstructed by template organic molecules. Therefore, the presence of a template in the zeolite channels acts as barrier in mass transfer between metal substrate and corrosion electrolyte, inducing a higher corrosion resistance [62].

Based on these considerations, hydrothermal synthesis of zeolite coatings with anti-corrosion performance remains a great and promising challenge, considering that this deposition strategy is able to simply obtain well-ordered and interconnected zeolite grain films [48,50].

### 3.3. Ionothermal In Situ Crystallization (I-InC) Method

In situ crystallization by ionothermal process is a relatively new method of zeolite synthesis, in which an ionic liquid is used in place of water as the solvent constituent (compared to in hydrothermal synthesis). An ionic liquid is a compound consisting of only ions, and usually has a melting temperature lower than 100 °C. The main advantage of using this technique is that the whole zeolite crystallization process can be managed at atmospheric pressure, due to the low vapor pressure of the ionic liquid [63], allowing avoidance of the under pressure autoclave step as required in hydrothermal synthesis.

Aluminophosphate zeolites have been effectively synthesized using the I-InC technique in order to obtain zeolite layers [42]. However, few articles have evidenced successful results on silicate based zeolite synthesized by ionic liquid in situ crystallization [64]. A relevant issue is the very limited solubility of silica precursor compounds in the commonly used ionic liquids [65]. However, one of the main problems in making zeolite coatings on metal supports is the pH of the synthesis solution, which is usually strongly alkaline. This makes it difficult to directly synthesize zeolite coatings on amphoteric metal substrates that undergo dissolution in an alkaline environment. The identification of synthesis conditions compatible with the electrochemical stability of the metal substrate during the in situ crystallization process is a key factor for the development of the technique, and for obtaining homogeneous, compact, and defect free coatings.

The non-aggressive synthesis conditions of high silica zeolite have allowed extension of the direct in situ coating deposition of these class of zeolite.

The first work, published in 2008, on anti-corrosion zeolite coatings synthesized by I-InC was proposed by Cai and co-workers [42]. They synthesized well-oriented zeolite coatings on AA 2024-T3 alloy. This aluminum alloy is most widely used for aerospace applications, although it is sensitive to corrosion phenomena due to its high copper content. Two different zeolite types were synthesized: ALPO-11 and SAPO-11 zeolites. ALPO-11 is a pure aluminophosphate zeolite with the AEL framework structure type. SAPO-11 has the same framework as ALPO-11, but it is characterized by silicon atoms that substitute some framework atoms in order to obtain a silicoaluminophosphate zeolite. The nature of the zeolite significantly influences the zeolite coating growth, and subsequently the anti-corrosion performances of the coating. The ALPO-11-based coating grows quickly and leads to the formation of a randomly structured coating with non-optimal barrier capabilities. Conversely, the SAPO-11 based coating crystallizes slowly, and leads to the growth of a well ordered and aligned zeolite layer on aluminum substrate. Better anti-corrosion performances were observed for the SAPO-11 coating, although the results were not as effective as observed for hydrothermal synthesis (Table 1). Most recently Yu et al. [43] investigated oriented ALPO-11 films as corrosion-resistant coatings, using a one-step ionothermal synthesis on aluminum substrates. Their results evidenced that the corrosion-resistant performance of the zeolite coating induced a corrosion current on the coated Al sample about three orders of magnitude lower than that uncoated one, reflecting a much more effective passive corrosion behavior. The results were more satisfactory than those previously reported by Cai [42], but further efforts are still needed in order to optimize the barrier properties of the zeolite coating compatibly with the deposition and growth process issues of the same.

### 3.4. Dry-Gel Conversion (DGC) Method

A limit of the hydrothermal approach is the need to carry out the synthesis under conditions of high temperature and pressure. At the same time, the need for an autoclave to finalize the crystallization of the zeolite imposes dimensional constraints on the components that can be coated, depending on the volume of the autoclave itself. A scale-up for large components would become economically disadvantageous. In this sense, ionothermal synthesis tends to minimize these problems, although it amplifies the difficulties in making homogeneous and compact zeolite coatings. However, an approach that has been developing in recent years and is receiving increasing interest from different research groups is represented by dry-gel conversion (DGC) method.

**Figure 4:**
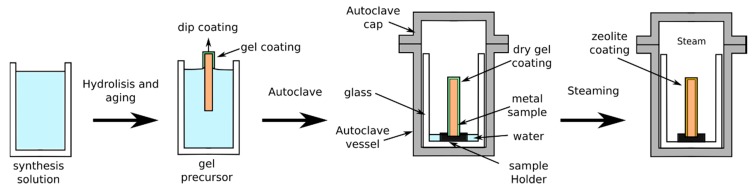
Scheme of dry-gel conversion synthesis process.

Differently to H-InC and I-InC synthesis, DGC synthesis involves crystallization of the zeolite layer in the presence of a steam environment, starting from a dry-gel precursor without the involvement of a liquid precursor [65,66]. The DGC method for obtaining zeolite layers on metal substrate can be considered constituted by three steps, as schemed in Figure 4:

1. First, a precursor sol, obtained by hydrolysis and aging of a diluted alcoholic silico/aluminate solution, is prepared.
2. Then, the so prepared precursor sol is deposited on the metal substrate (e.g., by spray or dip coating)
3. The coated metal substrate is dried and placed in an autoclave containing a low amount of distilled water to produce steam. The steaming phase activates the crystallization of a zeolite layer on the metal substrate.

This process, although constituted of several phases with respect to hydrothermal synthesis, has considerable advantages in terms of simplification of the crystallization process, but also less waste, less usage of templates, etc. Consequently, anti-corrosion coatings in zeolite can be applied more economically on a larger substrate. A further advantage of the DGC technique compared to the hydrothermal one is that the film thickness of an MFI film can be checked directly by the precursor amount deposited on the metal substrate before the steaming conversion process. These advantages have significantly stimulated the research activities in this field in order to obtain a barrier corrosion protective zeolite layer.

In Table 2, a brief summary of scientific work focused on dry-gel conversion zeolite coatings is reported. Analogously to H-InC and I-InC techniques (Table 1), the main information related to coatings typology, synthesized to evaluate their effectiveness as a protective coating against corrosion, is reported.

At first, in 2013, Changjean et al. [35] demonstrated the effectiveness of the steam-assisted conversion of pre-deposited sol-precursor film on an aluminum alloy as anti-corrosion zeolite coating. The DGC coating sample evidenced a reduction of the corrosion current of about 5 decades compared to bare metal. This anti-corrosion behavior of the zeolite layer has been attributed to the higher polarization resistance due to a dense, compact and non-permeable zeolite coating. The pores and channels in the prepared zeolite film were blocked by the structure directing agent. In the same year Pande et al. [58] have reported a very extensive work in which demonstrated an effective protective action of DGC ZSM-5 zeolite coatings in different acidic environmental conditions. Afterwards, other works were published evidencing the suitability of this deposition technique [8,50]. More recently, in 2018, Tsai et al. [36] realized a DGC coatings by using MFI zeolite showing its performance stability in the whole pH range of NaCl electrolyte solution.

**Figure 5:**
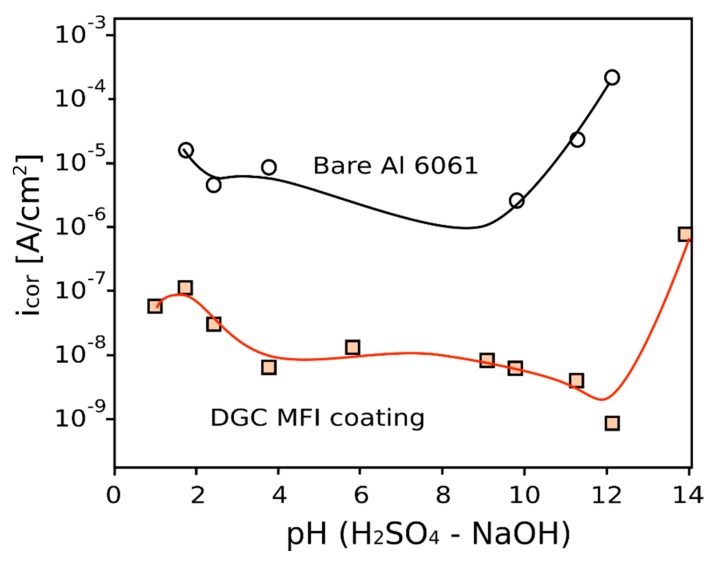
Corrosion rate of bare and DGC coated Al 6061 substrate at varying pH in H_2_SO_4_–NaOH solutions [36].

The high efficiency of DGC coating can be also related to a proper crystal morphology where zeolite nanocrystals are well packed compared to InC technique thus increasing the zeolite layer resistance [36]. More recently Tsai et al. [36] compared anti-corrosion performances of MFI coatings on AA6061 alloy obtained by in situ crystallization and dry-gel conversion, respectively (Figure 5). MFI zeolite coatings showed a significant corrosion resistance in the whole pH range, evidencing corrosion current five order of magnitude lower than the uncoated Al alloy in NaCl solution. Furthermore, they indicated that protection ability of zeolite coating is attributed to the increased film resistance associated with a dense and impermeable zeolite layer. In particular, by using the dry-gel conversion method as deposition technique was possible to favor the grown of high packing density zeolite layer with higher barrier action that than the in situ crystallization grown film.

**Figure 6:**
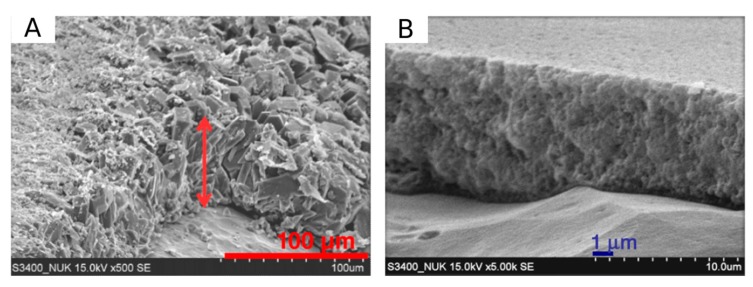
Cross-section SEM image of the zeolite coating produced using the (**A**) hydrothermal and (**B**) dry-gel-conversion process [35]. Reprinted with permission from Elsevier.

In Figure 6 a comparison of zeolite coating obtained by hydrothermal and dry-gel conversion method, obtained by Chengjean et al. [35], is reported. They evidenced that the zeolite coating obtained by hydrothermal method is a thick layer of randomly stacked zeolite crystals with some voids and heterogeneity. Although a sufficient dense packing with good crystal inter-growth takes place, it is plausible that more than one layer of crystals constitutes the zeolite layer favoring a not homogeneous and regular coating cross section structure and profile. Conversely, on zeolite coating synthesized by DGC method is characterized by a denser layer with a well packed zeolite nanocrystals. The authors also evidences that the coating thickness can be easily controlled by the gel precursor solution dipped on the metal sample before the steam-assisted conversion phase. However, it should be noted that the DGC coating, although compact and homogeneous along the whole thickness, shows a lower interaction with the zeolite-support interface than hydrothermal one. This can be related to the intrinsic difference between the two synthesis techniques. While in the in H-InC and I-InC, crystals germinate directly on the metal surface implying a strong chemical interaction at the interface, the crystallization process of the zeolite coating in the DGC technique occurs during the steaming phase after the dry-gel layer coated (e.g., dip coating procedure) the metal substrate.

Concerning to the denser and compact coating obtained by DGC process compared to the other in situ crystallization methods it is directly related to the smaller size of the zeolite nanocrystals that can be produced [35,67].

The production of spherical nanocrystals takes place, since the nucleation occurred in the gel precursor characterized by a high concentration of silica [68]. The packing of these spherical particles turns out to be more effective, the distance between the different nanocrystals is reduced and the coating becomes more dense compared to larger crystals and of prismatic shape as obtainable in the case of other in situ crystallization synthesis methods. The formation of a thicker and more compact zeolite film is a key element for stable electrochemical properties and high anti-corrosion performances.

### 3.5. Morphological Aspects of Corrosion Resistance of the Coatings

The corrosion resistance is significantly influenced by voids and cracks in the zeolite barrier coating [41]. A zeolite synthesis optimized to minimize the presence of defects, and to guarantee the growth of a highly homogeneous and compact coating, is key in obtaining an effective corrosion-resistant coating. Chau et al. [69] evidenced that the chemistry, structure, and morphology of the substrate material plays a relevant role in zeolite layer formation and growth of the zeolite crystals. At the same time, the voids and cracks are influenced and triggered by synthesis parameters and the crystal growth behavior of the coating. A good crystal inter-growth plays a relevant role in the coating anti-corrosion performance. At the same time, adhesion with the metal substrate is another parameter with critical relevance to the coating stability in severe environmental conditions.

**Figure 7:**
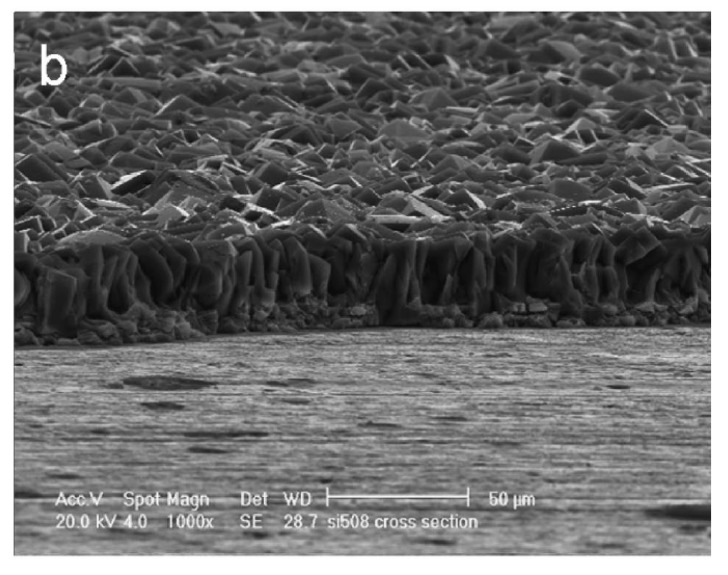
Cross-section view of an as-synthesized ZSM-5 zeolite coating on Al-2024-T3. The cross-sectional sample was prepared by etching away part of the film with hydrofluoric acid [29]. Reprinted with permission from Electrochemical Society, Inc.

In Figure 7, an example cross-section view of a zeolite coating synthesized by H-InC is reported. The coating is compact and well adhered to the substrate (Al 2024-T3). The inter-crystalline structure and interconnected growth of zeolite grains is visible. The coating thickness uniformly covers the metal substrate. For suitable and effective corrosion resistance of a polycrystalline zeolite coating, the InC needs to be performed in order to obtain an adherent, dense, and compact layer. No evidence of inter-crystal porosity and intra-crystal porosity should be observed [29]. A good intergrowth of zeolite grains is able to reduce the inter-crystal porosity [70], leaving trapped in the zeolite structure-directing agent (SDA) molecules [30] or synthetic products that have an inhibitory action against corrosion phenomena (e.g., amines [71] or amorphous silicates and aluminates [72]).

However, it is particularly difficult to obtain undamaged and crack-free coatings; correct optimization of the process parameters is required in order to fix these issues [47].

**Figure 8:**
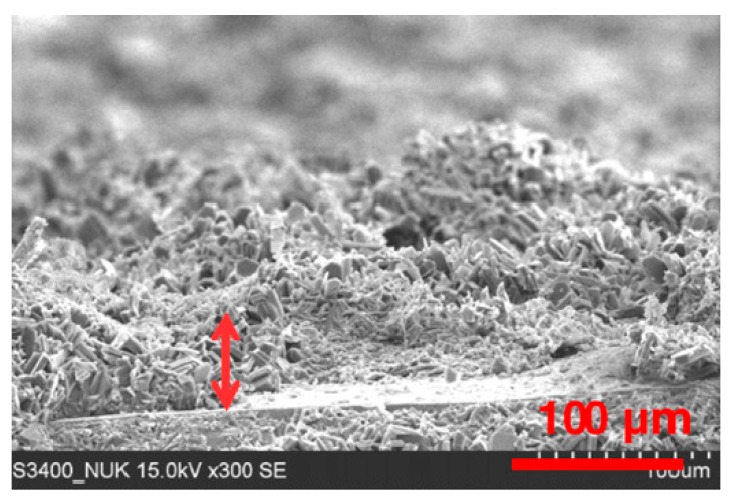
Cross-section SEM image of the zeolite coating produced by non-optimal hydrothermal synthesis [35]. Reprinted with permission from Elsevier.

Figure 8 shows a MFI zeolite coating obtained by 20 h of hydrothermal in situ crystallization synthesis, according to the procedure described in Reference [35]. The non-optimal process conditions led to the formation of a non-homogeneous coating with inhomogeneities in terms of interconnection of the crystalline grains and layer thickness. Furthermore, the presence of defects and voids can be seen randomly in the whole deposited layer. The combination of defects (e.g., cracks or voids) and low adhesion stimulated the formation of preferential pathways for water diffusion that reaches and accumulates easily at the metal coating interface, thus allowing activation of a premature failure mechanism of the protective barrier coatings [73].

## 4. Anti-Corrosion Aspects of Zeolite Coatings

### 4.1. Electrochemical Properties of the Zeolites

The anti-corrosion behavior of zeolite-based coatings can be partially related to the electrochemical properties of the zeolite itself. Although zeolites are electronic insulators, electronic transfer is at the basis of their electrochemical response, and different transfer mechanisms have been proposed [74,75,76]: (a) extra-zeolite electron transfer, (b) intra-zeolite electron transfer and (c) electron transfer to/from species located at the outermost boundaries of the zeolite particles.

(a) In the extra-zeolite electron transfer mechanism, redox species diffuse to a conductive electrode surface (e.g., a metal surface), where charge transfer occurs, after being initially ion-exchanged by the electrolyte cations.
(b) In the intra-zeolite electron transfer mechanism, charge transfer occurs via electron hopping between adjacent redox species located in the zeolite structure.
(c) The last mechanism involves two steps: in the first step, the electro-active species situated at the outer surface of the zeolite particles undergo electron transfer and then, in the second step, they act as mediators for the redox transformation of those located in the bulk of the solid (each step requires charge compensation by the electrolyte cations).

At the same time, it is important to evaluate the strong ionic nature of zeolites that is at the basis of their ionic conduction properties. The direct involvement of the ions of the zeolite framework in electrochemical reactions has been also reported in the literature. The whole redox process could be therefore governed by the diffusion of the electrolyte cations in the zeolite pore systems [77]. The destruction of the Al–O bond at an anodic potential close to 1 V and, on reversed scan, the extrusion of Al(OH), leading to the formation of small silica clusters, was proposed by Shi [78] during voltammetry of zeolite in poly(ethylene oxide) oligomer.

It is therefore clear that direct involvement of the zeolite film is expected during anodic and cathodic polarization, but since no clear hypothesis of the reactions occurring is yet available, a more deep investigation must be carried out, where particular attention is to be given to the interactions and exchange processes involving electrolyte ions.

### 4.2. Barrier Mechanisms of Zeolite Coatings

The anti-corrosion performance of zeolite film can be due at first to a barrier property, due to the reduced substrate area available for anodic and cathodic reaction.

The barrier action increases if the intra-crystal and intra-crystal porosity is reduced. For the former, a good packing of zeolite crystals during growth reduces the inter-crystalline porosity and therefore increases the resistance to mass diffusion along the thickness of the coating, thus enhancing its protective action [32]. Furthermore it has been found that the intra-crystal porosity can be reduced by keeping entrapped in the zeolite framework the structure-directing-agent (SDA) molecules [30] or synthetic products that have an inhibitory action against corrosion phenomena (e.g., amines [47,71] or amorphous silicates and aluminates [72]).

At the same time, an active behavior of the zeolite layer, supporting the protective performance of the coating, can be hypothesized. In particular, according to Figure 5, a significant different i_cor_ can be observed in alkaline environments. Calabrese at al. [47] related this effective and efficient behavior at high pH to the growth of a thick and compact layer of aluminate corrosion products, interconnected to the zeolitic framework of the coating. The proposed precipitated mechanism is triggered by the presence of cations in the electrolyte solution (from dissociation of hydroxides present in alkaline electrolyte), which react with the corrosion products of the metal substrate. For example, in saturated Ca(OH)_2_ solution, an insoluble surface layer of hydrated calcium aluminate Ca(AlO_2_)_2_·zH_2_O forms, constituted by the reaction of aluminate ions AlO_2_^−^, which are present at pH values above 12, with Ca^2+^ ions [79] according to the following reactions:

Al + 2H_2_O → AlO_2_^−^ + 4H^+^ + 2e^−^ (2)

O_2_ + 2H_2_O + 4e^−^ → 4OH^−^ (3)

2 AlO_2_^−^ + Ca^2+^ +H_2_O → Ca(AlO_2_)_2_↓ + H_2_O (4)

Thus, corrosion will cease to evolve. Furthermore, the presence of zeolite, which in this alkaline solution exchanges sodium with calcium cations, could favor the nucleation and grown of calcium aluminate on the sample surface.

Furthermore, the good anti-corrosion performances of zeolite based coatings could also be related to an inhibitory action. Based on this idea, a scheme of corrosion protection of defected areas of zeolite based coatings was proposed recently in Reference [80].

**Figure 9:**
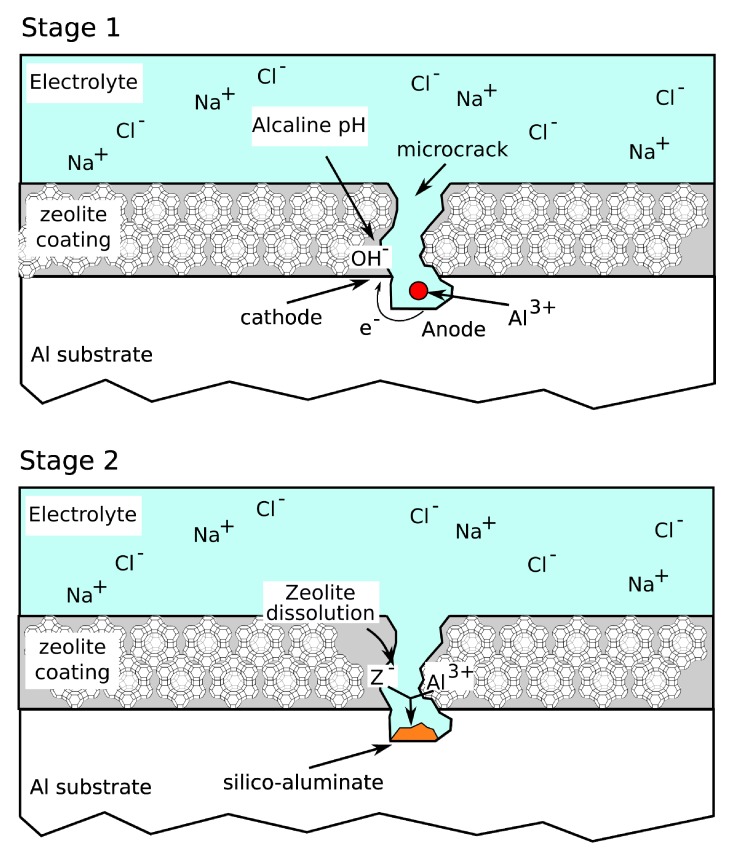
Scheme of corrosion protection on defected areas of zeolite based coatings.

When redox reactions occur on defected areas of a zeolite based coating, the cathodic reaction locally favors an increase in the pH of the solution (stage 1, Figure 9). The OH groups could be adsorbed by the functional species (> Si−OH and > Al−OH) present on the zeolite framework, both on channels (internal surface) and terraces (external surface). Thus, the interaction with the hydroxyl ions of the zeolite leads to the formation of > Si−O and > Al–O, which then could weaken the Si−O− and Al−O− bonds in the lattice of the surface and therefore, favor the detachment of zeolite framework portions [81]. The > Si−O and > Al−O groups are able to react with the metal cations generated due to dissolution phenomena in the anodic site (e.g., Al^3+^ ions), forming a passive silicate layer [82] that preserves the metal substrate from further corrosion processes (stage 2, Figure 9). Thus, the anti-corrosion performance of the protective layer is stable, due to the passivation phenomenon of the anodic areas until the middle period [80].

## 5. Anti-Corrosion Efficiency of In Situ Crystallization Zeolite Coatings

The extent of the corrosion inhibition induced by zeolite coating deposition was evaluated using an efficiency index (EI) defined according to Equation (5).

EI= log (i_cor(coated)_/i_cor(bare)_) (5)

**Figure 10:**
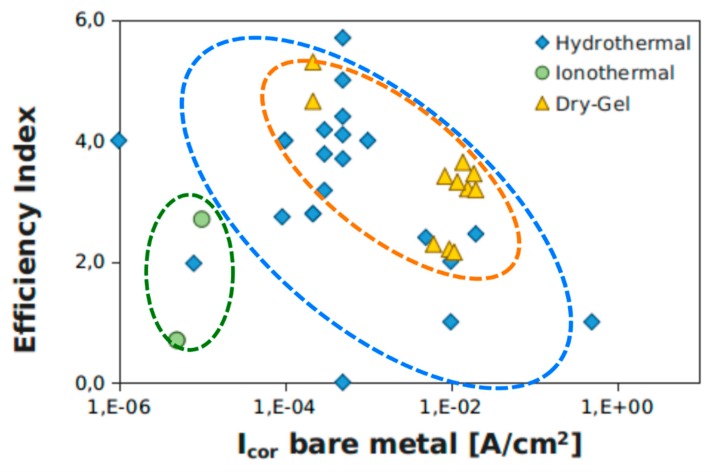
Efficiency index versus corrosion current of bare metal support.

EI is defined as the logarithm on base ten of the ratio between the corrosion currents of the zeolite coating and bare substrate respectively. According to this definition, EI identifies the current decade’s shift of i_cor_ in zeolite layers. The EI parameter was determined for all experimental configurations identified in Table 1 and Table 2. The results are summarized in Figure 10, where the efficiency index versus corrosion current of bare metal is plotted. Data were clustered based on the crystallization technique used for zeolite coating deposition: hydrothermal, ionothermal, and dry-gel conversion methods.

Zeolite coatings obtained by the ionothermal crystallization method evidenced a lower efficiency index. This indicates that this deposition technique, although it favors the zeolite crystallization process at atmospheric pressure, does not give a zeolite layer with efficient barrier capabilities. The coating is probably not dense and compact enough to guarantee effective anti-corrosion performance. The presence of local heterogeneity or defects favors the formation of localized preferential pathways for water diffusion, which induces an irrelevant efficiency index to corrosion protection of this zeolite layer. With regard to hydrothermal synthesis, a wide dispersion of EI data was found. This wide heterogeneity of results is attributable to the relative difficulty of managing the synthesis process parameters during both the aging of the precursor solution and the hydrothermal crystallization in autoclaves under pressure and temperature. The definition of erroneous process parameters implies a coating with large defects and low cohesion. However, for optimized hydrothermal synthesis processes, the coatings have a very good EI parameter, indicating strong adhesion to the substrate and an excellent interconnection between the zeolite crystals, characterized by low inter-crystal and intra-crystal porosity. The DGC technique highlighted the most interesting results. All the polarization tests found in the literature showed a good efficiency index, indicating good protective properties of the polycrystalline zeolite layer. Furthermore, the synthesis technique is relatively easy, as confirmed by the limited dispersion of the data population in the graph of Figure 2. The dry-gel cluster (dotted yellow ellipse) is located inside the hydrothermal cluster (dotted blue ellipse), with the values placed at the upper threshold of the hydrothermal technique coatings data. This certainly places particular attention on the DGC technique for further future development, and improvement of the synthesis and optimization of coatings with anti-corrosive action on metal supports.

## 6. Functional Anti-Corrosion Coatings

A relevant aspect that improves the anti-corrosion capabilities of zeolite based coatings is the opportunity to combine barrier with active protection, obtaining so called smart coatings.

Chromates are widely used in the coating industry as inhibitory pigments to offer high durability and self-repairing capability on paints. A green and sustainable alternative, able to avoid chromate use, is the use of ion-exchange micro-porous and meso-porous fillers that act as nano-carriers/containers of the inhibitory agent [83,84,85]

Considering that zeolites are ion-exchange materials, it is possible to prepare active pigments by exchanging, for example, sodium cations present in zeolites with other cations that can instead provide inhibiting and self-healing properties to anti-corrosive coatings. The zeolite filler could be suitably doped with an inhibitor agent that can be released during corrosion, triggering the corrosion processes.

Zeolite containers act as active capsules, having entrapped the healing agent. If the coating is locally damaged, the active capsule releases the inhibitor entrapped in its structure, activating the healing process [86]. The coating acquires an additional functional property, that if the inhibiting action is active, a self-repairing coating can be obtained [87,88]. For example, when a scratch is generated on chromate coatings, a spontaneous repassivation of the active surface occurs. In order to offer such self-healing benefits, an active, green, and safe pigment must be added to retain the same chromate capabilities. In such a context, ion exchange agents have been shown to be effective and suitable compounds that can be encapsulated in the zeolite structure to offer inhibiting action to the smart coating. In the past zeolite has been used as nano-container for antimicrobial agents, like silver ions, that gradually releases the active antimicrobial ion into the environment [89,90,91].

The combination of multi-functional capabilities, such as antimicrobial properties and corrosion resistance, coupled with the opportunity to deposit the layer on several structural metal substrates, makes zeolite coatings advantageous over the current sol-gel coatings in specific industrial applications where critical or unconventional environmental conditions are required (e.g., adsorption-based heat pumps or spacecraft applications) [92,93,94].

Concerning anti-corrosive paints, Deya et al. [81] investigated a natural zeolite rock modified by ionic exchange with molybdenyl ions, finding it to be effectively protect steel alloy from corrosion. When water and ions penetrated the coating, the ions were exchanged with molybdenyl cations, which then hydrolyzed to generate molybdate anions. Molybdates are compounds with effective inhibitor capability on steel alloy, by forming a layer of ferrous molybdate [95,96].

Furthermore, good, active anti-corrosion performances were observed by Dias et al. [34,97] when using NaX zeolite filler exchanged with cerium cations. They observed that the precipitation of cerium hydroxide, Ce(OH)_3_, on the cathodic areas of the substrate was the key factor in inhibition of corrosion on the metal substrate. Ferrer et al. [98] also developed a zeolite based composite coating with cerium/diethyldithiocarbamate double-doped NaY zeolite as an efficient corrosion inhibitor for AA2024-T3. Similar results were obtained using lantanium ions [99]. The self-repairing mechanism could involve a progressive release of molybdate and lanthanum ions from the modified zeolite that induces the formation of a Mo-Na-La compound on intermetallic particles with inhibitory properties. The results confirm the potential effectiveness of this approach for suitable environmentally friendly and active corrosion protection.

Concerning the healing mechanism offered by zeolite modified capsules, it can be schemed as following: At first, due to the interaction of the coating with the aggressive electrolyte, several micro-electrochemical cells are generated on the surface of the coating. Each micro-cell is constituted of cathodic and anodic areas where oxygen reduction reactions and dissolution of the substrate take place, respectively. The oxygen reduction reaction stimulates a local modification of pH, which easily increases above pH 8. This favors the formation of oxides, such as cerium oxide, according to Equation (6) [100].

(6) $${\mathrm{Ce}}^{3+}+3{\mathrm{OH}}^{-}\to \mathrm{Ce}{\left(\mathrm{OH}\right)}_{3}\overset{1/2H_{2}O_{2}}{\to }\mathrm{Ce}{\left(\mathrm{OH}\right)}_{4}\to {\mathrm{CeO}}_{2}\cdot 2H_{2}O$$

**Figure 11:**
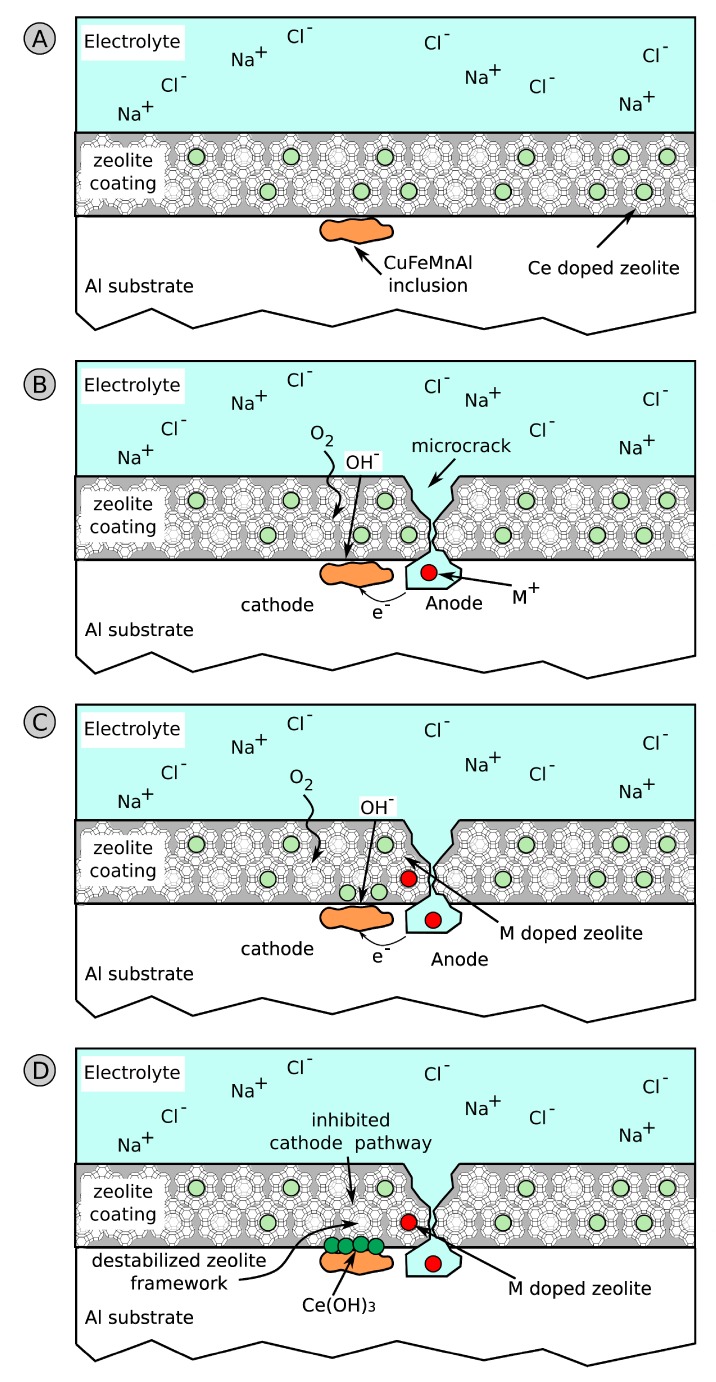
Scheme of the self-healing process of Ce doped zeolite coatings. (**A**) Zeolite coated substrate. (**B**) Intermetallic inclusion contribute on anode/cathode reactions. (**C**) Ion exchange process and cathodic zeolite destabilization for the release of Ce^3+^. (**D**) Precipitation of Ce oxide/hydroxide on intermetallic inclusion.

A schematic diagram of the process is reported in Figure 11.

Due to a scratch or local defect on the coating, a solution flow through the coating takes place, activating local corrosion of the substrate. Anodic and cathodic areas are created (Figure 11B). As evidenced in the figure, the cathodic inclusions in the metal alloy play a relevant role in the corrosion inhibition action of zeolite [97].

As discussed in Reference [34], for zeolite microcapsules there is an effective adsorption of Cu^2+^ and Cl^−^ on the Ce-modified zeolite container. The zeolite self-healing capabilities are directly related to the amount of Al atoms in the structure, and the type of exchanged cations. Zeolite exchange with metal cations can be considered as a solid Lewis acid–base reaction, where the Lewis acidity is due to extra cations and the Lewis basicity to the lattice oxygen. This allows ion adsorption in Brønsted acid sites of the zeolite lattice, since these sites are able to donate a proton to an incoming basic molecule [101].

Due to the adsorption of metal cations in order to maintain charge stability, the release of Ce^3+^ ions, by ion-exchange process, from the active zeolite to the aggressive electrolyte occurs.

At the same time, coupled to the ion exchange phenomenon, cerium ion release is favored by high local pH in cathodic areas. The interaction of hydroxyl ions with the channels (internal surface) and terraces (external surface) of the zeolite structure weakens the Si−O− and Al−O− bonds in the lattice of the surface, and favors instability of the zeolite framework [81] and the local release of the cations encapsulated in the structure.

Therefore, in correspondence of the intermetallic cathodic inclusions, there is an enrichment of Ce^3+^ ions (Figure 11C), which precipitate in the form of cerium hydroxide, Ce(OH)_3_ (Figure 11D). The cerium hydroxide thus formed has a protective action against the corrosion phenomenon precisely at the incipient corroded area, thus providing self-repair of the damage that is originating. Furthermore, the cerium hydroxide, due to the alkaline environment in the cathodic area of the inclusion, can further evolve into cerium oxide, according to the previous reported Equation (6), with an increase of the passivating action. The result is the slowing of the cathodic reaction, thus reducing further evolution of the corrosive phenomenon [102].

**Figure 12:**
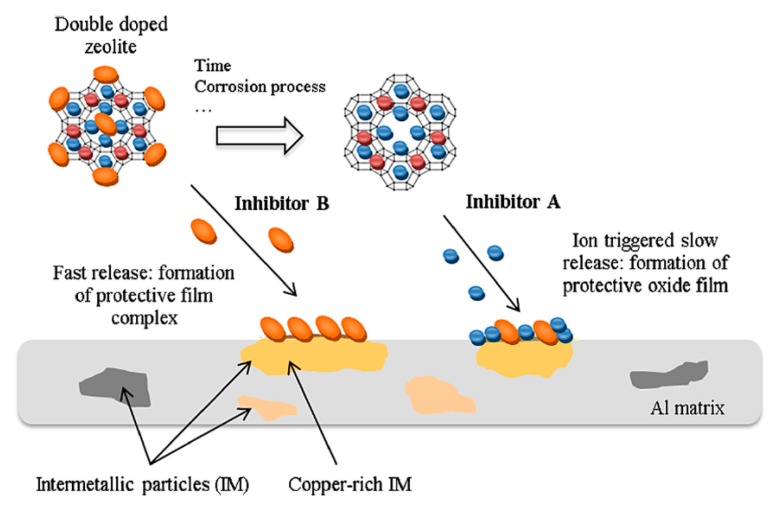
Scheme of the self-healing process of double doped zeolite on AA2024 metal substrate [98]. Reprinted with permission from Elsevier.

Based on this approach, Ferrer et al. [98] proposed zeolite as nano-container for two classes of inhibitor, proposing a double doping approach of zeolite based coatings. A scheme that summarizes the double doping concept on zeolite based coatings is reported in Figure 12.

The zeolite container is filled with two different corrosion inhibitors, based on two protection approaches. When the zeolite coating is exposed in the aggressive electrolyte solution, a faster inhibitor is released (inhibitor B in Figure 12). This phenomenon offers a rapid corrosion protection of the metal substrate in the short–medium period through surface complexation. Afterward, the second inhibitor (inhibitor A in Figure 12) remains hosted in the zeolite container, favoring a progressive release over medium–long time, triggered by ion exchange when the corrosion process advances. The double-doping of zeolite becomes effective when a synergistic effect between the two selected inhibitors takes place, guaranteeing suitable protection over long periods thanks to the controlled and progressive release process. Figure 12 summarizes the two-step release approach from a cerium/diethyldithiocarbamate double-doped NaY zeolite. This approach is more interesting, and it is based on potentially synergistic behavior by the coupled doped zeolite crystals. When released, the inhibitors act mainly at cathodic sites. At first, the corrosion protection is related to the rapid release of the diethyldithiocarbamate (DEDTC) that is adsorbed on the outer surface of the zeolite crystal. The relevant affinity of DEDTC to copper on the AA2024 aluminum alloy, combined with the ion exchange with the Cl^−^ ions in solution, could be considered to be the main mechanisms for the surface corrosion protection. Afterwards, cerium released from the zeolite occurs more slowly through an ion exchange mechanism between the cations from solution and the cerium in the zeolite. The cerium has a higher inhibitory action and is able to offer a more effective protection over a long time. Furthermore, cerium hydroxide/oxide precipitates constitute a compact protective layer on copper-rich intermetallic particles, thus preventing further cathodic activity on these sites [103] and inducing an additional effect able to improve corrosion protection of the metal substrate.

Analogously, Caprì et al. [104] highlighted that the surface adsorption process plays a key role in zeolite doping with inhibitors. They tested the self-repair action of a SAPo-34 zeolite coating. SAPO-34 is an inert-exchange zeolite, and its carrier action to inhibitors cannot be carried out by ion exchange. The results showed that the cerium was not hosted inside the zeolite crystal, but remained adsorbed on the external zeolite surface. However, the inhibiting action allowed a durable coating with stable electrochemical behavior up to a year of immersion in a solution of 3.5% NaCl.

These results further enhance the potential applicability of zeolites as carriers for corrosion inhibitors, highlighting their combination both for ionic and organic inhibitors, providing possible multi-functional and long-lasting protective action.

## 7. Conclusions

The present manuscript provided a critical review on anti-corrosion behavior of zeolite coatings obtained by in situ crystallization methods on several metal alloys. A brief description of zeolite direct synthesis approaches was reported, in order to better relate the anti-corrosion performances to the microstructure and coating morphology, strictly related to the in situ crystallization techniques used. The dissertation on this topic evidenced that suitable and effective barrier protective capabilities of zeolite based coatings deposited on different metal substrates such as and stainless steel, aluminum, titanium, or magnesium alloys can be obtained, evidencing a corrosion current of the coated samples up to five orders of magnitude lower than bare metal substrate. In particular, more recently, very promising results have been evidenced by zeolite coating obtained by dry-gel conversion method, concerning both anti-corrosion performances and zeolite coating microstructure. Furthermore, the literature results highlight that the anti-corrosion performances of the coatings are stable in multiple environmental conditions, ranging from chloride environments up to acidic or strongly alkaline electrolytes. Finally, the electrochemical behavior was assessed, detailing some barrier mechanisms of zeolite coatings proposed in the literature, focusing finally attention on the self-repairing action that the zeolite coatings can acquire if opportunely doped.

## 8. Future Trends

Zeolite-based coatings have great potential to become effective and efficient eco-sustainable anticorrosion coatings, e.g., as suitable alternatives to traditional chromium-based conversion layers. Development of a versatile deposition technique is a key factor that will allow these coatings to be applied to multiple structures, including those characterized by complex geometries. The possibility of depositing coatings without heavy process conditions as in the DGC method is a stimulus for further research and development activities, aimed at future use in the surface finishing industry for anti-corrosion applications. The different deposition techniques investigated in this review, although effective in terms of barrier action and corrosion resistance, in fact require further improvement to optimize the synthesis layout by providing a simple and versatile deposition process for different zeolite coatings, thus further expanding the application fields.

In such a context, the possibility of conferring multi-functional properties on zeolitic coating represents further added application value of great interest. Self-repair, antimicrobial, or hydrophobic surface capabilities are possible configurations to be integrated with the barrier anti-corrosion action of the coating. Multi-functional smart coatings are a very attractive research area, and represent a field of considerable interest and investments for the future. For example, smart coatings can provide both the release of an on-demand corrosion inhibitor and other integrative functions such as conductivity, self-diagnosis, or viracolor, expanding interest in the coatings/sensors applications. This phase of future engineering will be stimulated by the consolidation of knowledge of nano-materials, benefiting and accelerating the industrialization of zeolite based coatings.

## Figures and Tables

**Table 1:** Brief summary of the literature review of anti-corrosion performances of zeolite-based coatings obtained by the in situ crystallization technique.

*Authors*	*Ref.*	*Year*	*Method*	*Zeolite*	*Substrate*	*Electrolyte*	*I_cor_* [A/cm^2^]		*|Z| 0.01* Hz [Ohm*cm^2^]	
							*Coating*	*Substrate*	*Coating*	*Substrate*
Cheng et al.	[29]	2001	Hydrothermal	ZSM-5	Al 2024-T3	0.5 M H_2_SO_4_ 0.5 M NaCl/HCl	~1 × 10^−10^ ~1 × 10^−10^	~1 × 10^−6^ ~1 × 10^−2^	~1 × 10^9^ ~1 × 10^9^	~1 × 10^5^ ~1 × 10^2^
Mitra et al.	[41]	2002	Hydrothermal	MTW	Al 2024-T3	0.5 M H_2_SO_4_	~2 × 10^−7^	~3 × 10^−4^		
Mitra et al.	[41]	2002	Hydrothermal	MTW BEA	Al 6061-T4	0.5 M H_2_SO_4_	~2 × 10^−8^ ~5 × 10^−8^	~3 × 10^−4^		
Mitra et al.	[41]	2002	Hydrothermal	MTW	Al 2024-T3	0.1 M NaOH	~1 × 10^−7^	~1 × 10^−3^		
Mitra et al.	[41]	2002	Hydrothermal	MTW BEA	Al 6061-T4	0.1 M NaOH	~2 × 10^−8^ ~4 × 10^−8^	~5 × 10^−4^		
Mitra et al.	[41]	2002	Hydrothermal	MTW MFI BEA	AISI 304	0.5 M H_2_SO_4_	~1 × 10^−7^ ~1 × 10^−9^ ~5 × 10^−9^	~5 × 10^−4^		
Bedi et al.	[46]	2009	Hydrothermal	MFI	Ti6Al4V	0.856 M NaCl 0.856 M NaCl/HCl	8.6 × 10^−8^ 1.7 × 10^−7^	8 × 10^−6^ 9.3 × 10^−5^		
Bonaccorsi et al.	[32]	2011	Hydrothermal	Y	AISI 304	5% NaCl Ca(OH)_2_ sat	~1 × 10^−4^ ~5 × 10^−3^	~1 × 10^−2^ ~2 × 10^−3^		
Bonaccorsi et al.	[32]	2011	Hydrothermal	Y	Al 6061	5% NaCl Ca(OH)_2_ sat	~2 × 10^−5^ ~5 × 10^−2^	~5 × 10^−3^ ~5 × 10^−1^		
Dong et al.	[48]	2012	Hydrothermal	Silicalite-1	AA 1060	0.5 M H_2_SO_4_ 0.5 M NaCl	1 × 10^−8^ 1 × 10^−8^	1 × 10^−4^ 1 × 10^−4^		
Banerjee et al.	[49]	2014	Hydrothermal	ZSM-5	AZ91D	0.1 M NaCl	1 × 10^−3^	1 × 10^−2^	4.5 × 10^4^	8 × 10^3^
Calabrese et al.	[47]	2014	Hydrothermal	Y	Al 6061	3.5% NaCl Ca(OH)_2_ sat	~5 × 10^−4^ ~7 × 10^−5^	~5 × 10^−4^ ~2 × 10^−2^		
Huang et al.	[50]	2015	Hydrothermal	MFI	Al 6061-T6	3.5% NaCl	1 × 10^−6.45^	1 × 10^−3.66^	1.7 × 10^3^	2.7 × 10^2^
Tsai et al.	[36]	2018	Hydrothermal	MFI	Al 6061-T6	3.5% NaCl	1 × 10^−6.45^	1 × 10^−3.66^	1.7 × 10^3^	2.7 × 10^2^
Cai et al.	[42]	2008	Ionothermal	SAPO-11 ALPO-11	Al 2024-T3	0.1 M NaCl	~1 × 10^−6^ ~1 × 10^−6^	~5 × 10^−6^		
Yu et al.	[43]	2018	Ionothermal	ALPO-11	Aluminum	0.1 M NaCl	~2 × 10^−8^	~1 × 10^−5^		

**Table 2:** Brief summary of literature review of anti-corrosion performances of zeolite-based coatings obtained by the dry-gel conversion technique.

*Authors*	*Ref.*	*Year*	*Method*	*Zeolite*	*Substrate*	*Electrolyte*	*I_cor_* [A/cm^2^]		*|Z| 0.01* Hz [Ohm∗cm^2^]	
							*Coating*	*Substrate*	*Coating*	*Substrate*
Changjean et al.	[35]	2013	Dry gel	MFI	Al 6061-T6	3.5% NaCl	1 × 10^−8.96^	1 × 10^−3.66^		
Pande et al.	[58]	2013	Dry gel	ZSM-5	Mild Steel	0.5M HCl 1.0M HCl 1.5M HCl	3.3 × 10^−6^ 1.0 × 10^−5^ 1.3 × 10^−5^	8.5 × 10^−3^ 1.6 × 10^−2^ 2.0 × 10^−2^		
Pande et al.	[58]	2013	Dry gel	ZSM-5	Mild Steel	0.5M H_2_SO_4_ 1.0M H_2_SO_4_ 1.5M H_2_SO_4_	3.2 × 10^−6^ 6.7 × 10^−6^ 5.8 × 10^−6^	1.4 × 10^−2^ 1.9 × 10^−2^ 1.2 × 10^−2^		
Pande et al.	[58]	2013	Dry gel	ZSM-5	Mild Steel	0.5M H_3_PO_4_ 1.0M H_3_PO_4_ 1.5M H_3_PO_4_	3.2 × 10^−5^ 6.0 × 10^−5^ 7.7 × 10^−5^	6.2 × 10^−3^ 9.6 × 10^−3^ 1.1 × 10^−2^		
Al-Subaie et al.	[8]	2015	Dry gel	Beta	Carbon Steel	3.0% NaCl 0.1M H_2_SO_4_ 0.1M NaOH 3.5% NaCl	1 × 10^−3.54^ 1 × 10^−1.41^ 1 × 10^−3.83^			
Huang et al.	[50]	2015	Dry gel	MFI	Al 6061-T6	3.5% NaCl	1 × 10^−8.31^	1 × 10^−3.66^	2.5 × 10^4^	2.7 × 10^2^
Tsai et al.	[36]	2018	Dry gel	MFI	Al 6061-T6	3.5% NaCl	1 × 10^−8.31^	1 × 10^−3.66^	2.5 × 10^4^	2.7 × 10^2^
